# CEPHEUS SA: a South African survey on the under-treatment of hypercholesterolaemia

**DOI:** 10.5830/CVJA-2011-044

**Published:** 2011-10

**Authors:** Frederick Johan Raal, Colin Schamroth, Dirk Blom, Jan Marx, Mangoo Rajput, Matthias Haus, Razia Hussain, Fatima Cassim, Michelle Nortjé, Guy Vandenhoven, Anne-Marie Temmerman

**Affiliations:** Department of Medicine, Charlotte Maxeke Johannesburg Academic Hospital, Johannesburg, South Africa; Milpark Hospital, Johannesburg, South Africa; Health Science Faculty, University of Cape Town, Cape Town, South Africa; Cardiology Research, Universitas Private Hospital, Bloemfontein, South Africa; RK Khan Hospital, Durban, South Africa; AstraZeneca, South Africa; AstraZeneca, South Africa; AstraZeneca, South Africa; AstraZeneca, South Africa; AstraZeneca, Belgium; AstraZeneca, Belgium

**Keywords:** hypercholesterolaemia, LDL-C, lipid-lowering drugs, statins, under-treatment

## Abstract

**Aim:**

The aim of the CEntralised Pan-South African survey on tHE Under-treatment of hypercholeSterolaemia (CEPHEUS SA) was to evaluate the current use and efficacy of lipid-lowering drugs (LLDs), and to identify possible patient and physician characteristics associated with failure, if any, to achieve low-density lipoprotein cholesterol (LDL-C) targets.

**Methods:**

The survey was conducted in 69 study centres in South Africa and recruited consecutive consenting patients who had been prescribed LLDs for at least three months. One visit was scheduled for data collection, including fasting plasma lipid and glucose levels. Physicians and patients completed questionnaires regarding their knowledge, awareness and perceptions of hypercholesterolaemia and the treatment thereof.

**Results:**

Of the 3 001 patients recruited, 2 996 were included in the final analyses. The mean age was 59.4 years, and 47.5% were female. Only 60.5 and 52.3% of patients on LLDs for at least three months achieved the LDL-C target recommended by the NCEP ATP III/2004 updated NCEP ATP III and the Fourth JETF/South African guidelines, respectively. Being male, older than 40 years, falling into the lower-risk categories, compliance with the medication regimen, and patient knowledge that the LDL-C goal had been reached, were associated with the highest probability of attaining LDL-C goals.

**Conclusion:**

The results of this survey highlight the sub-optimal lipid control achieved in many South African patients taking lipid-lowering therapy.

## Abstract

Cardiovascular disease (CVD), of which coronary heart disease (CHD) is the commonest manifestation, is the leading cause of mortality in adults in most European countries.^1^ Multiple lines of evidence (epidemiological, clinical and interventional) confirm that elevated levels of low-density lipoprotein cholesterol (LDL-C) are associated with increased cardiovascular risk and that lowering LDL-C reduces cardiovascular risk. This relationship between LDL-C and cardiovascular risk thus far holds true for almost all populations investigated.[Bibr R02]–4

Several authorities, such as the Joint European Task Force (JETF) and the United States (US) National Cholesterol Educational Program Adult Treatment Panel III (NCEP ATP III) have developed clinical guidelines for the management of CVD risk, as there are extensive data showing that modification of risk factors can delay the development of CHD or prevent recurrent events in those with CVD at baseline.^5^–^8^ Over time, these guidelines have proposed progressively lower targets for the cardiovascular risk factors, on the basis of clinical study evidence demonstrating that cardiovascular risk is reduced further by more rigorous risk factor control.^5^–^8^

Currently, all guidelines identify LDL-C as the primary target of cholesterol-lowering therapy, and recommend LDL-C goals based on the risk category of the individual patient.^7^,^8^ For many patients who are at low risk for future CVD events, non-pharmacological interventions such as diet, exercise and smoking cessation may be adequate risk-reduction strategies. However, patients with high cardiovascular risk generally require lipid-lowering drug therapy to reduce their risk adequately.

Despite the significant increase over the last decade in the number of patients treated with LLDs, surveys in Europe^9^–^13^ as well as in the US^14^,^15^ among individuals taking LLDs have shown that a relatively small proportion of patients achieve their target lipid levels. This is particularly the case when CVD risk is high and the lipid targets are set lower.

The CEntralised Pan-South African survey on tHE Undertreatment of hypercholeSterolaemia (CEPHEUS SA) was initiated to detect and quantify the degree of under-treatment of hypercholesterolaemia in South Africa, and to identify positive and negative determinants for being at LDL-C targets. This non-interventional study also investigated patient and physician characteristics as well as the attitudes of these two groups towards the management of hypercholesterolaemia.

## Methods

This non-interventional study was conducted between November 2009 and April 2010 in 69 study centres (both private and public sectors) in South Africa. Male and female patients aged 18 years or older who had been receiving LLDs for at least three months (without dose adjustments for at least six weeks) were eligible. Patients who came for their regular scheduled visit to the doctor/clinic were consecutively invited to participate in the survey. Patients who agreed to participate provided written informed consent. There were no exclusion criteria apart from unwillingness or inability to provide informed consent.

The final protocol was approved by the appropriate ethics committees as well as the South African Medicine’s Control Council. The survey was performed in accordance with the Declaration of Helsinki and the International Conference on Harmonisation of Good Clinical Practice guidelines.

CEPHEUS SA was a single-visit study. Before any patients were recruited, each investigator completed a questionnaire on his/her experience and perception of the management of hypercholesterolaemia, as seen in his/her patients. The investigators were asked to indicate their general attitude towards the diagnosis of hypercholesterolaemia, their perception of existing guidelines and goals, as well as their knowledge on the treatment of dyslipidaemia.

Before being assessed by the investigator, patients completed a questionnaire about their awareness and perceptions of hypercholesterolaemia, their current LLD regimen, and their compliance with the treatment.

For each patient, the investigator completed a patient record form, which included information on the patient’s demographics, current LLD treatment, and reason for initiating LLD treatment. The investigator also recorded the presence of known cardiovascular risk factors such as smoking, diabetes, family history of premature CHD (defined as definite myocardial infarction or sudden death before 55 years of age in father or other male first-degree relative, or before 65 years of age in mother or other female first-degree relative),^5^,^7^ arterial hypertension (defined as blood pressure ≥ 140/≥ 90 mmHg or current use of antihypertensive medication), and cardiovascular medical history. Investigators were specifically asked to identify patients in whom the primary reason for starting LLD was a diagnosis of familial hypercholesterolaemia.

Physical examination by the investigator was limited to measurement of height, weight, waist circumference and blood pressure. A fasting blood sample was drawn to evaluate the serum lipid profile [including measurement of apolipoprotein (Apo) AI and Apo B] and glucose level. Blood samples were collected in three tubes [5 ml in a gel tube, 2 ml in a fluoride tube and 2 ml in an ethylenediaminetetra-acetic acid (EDTA) tube] using materials provided by the central laboratory. Laboratory analyses were performed at Quintiles Laboratories, South Africa. Glucose, total cholesterol, HDL-C and triglyceride levels were analysed by the Roche BMD method. LDL-C (direct) was quantified by colorimetry, and Apo AI and Apo B were evaluated by the Roche Tinaquant method.

Normal weight was defined as a body mass index (BMI) < 25 kg/m^2^, overweight as a BMI > 25 and < 30 kg/m^2^, and obesity as a BMI > 30 kg/m^2^. Metabolic syndrome was defined as the presence of three or more of the following:^16^,^17^ abdominal obesity with a waist circumference of > 94 cm for men or > 80 cm for women; plasma triglyceride levels ≥ 1.7 mmol/l; a high-density lipoprotein cholesterol (HDL-C) < 1.0 mmol/l for men or < 1.3 mmol/l for women; blood pressure ≥ 130/≥ 85 mmHg; and a fasting glucose level ≥ 5.6 mmol/l.

## Study endpoints

The primary endpoint was the percentage of patients who achieved the LDL-C goals according to the NCEP ATP III/2004 updated NCEP ATP III guidelines,^6^ or the Fourth JETF7/South African guidelines which were current at the time the CEPHEUS SA study was conducted in South Africa (Table 1).

**Table 1.** LDL-C Goal Achievement Targets

*Risk category*	*NCEP ATP III/2004 NCEP ATP III*	*European*	*South African*
High risk			
Current goal (mmol/l)	< 2.6	< 2.5	< 2.5
Optional goal (mmol/l)	< 1.8	< 2.0	–
Medium/low risk			
Current goal (mmol/l)	< 3.4	< 3.0	< 3.0

Achievement of the LDL-C goal was also evaluated according to (1) the presence or absence of the metabolic syndrome; (2) primary versus secondary prevention; (3) demographic variables, (4) CVD risk factors, (5) class of lipid-lowering agent and (6) patient and physician determinants. In addition, the percentage of patients achieving the non-HDL-C goal of < 3.36 mmol/l according to the NCEP ATP III/2004 updated NCEP ATP III guidelines was also evaluated in patients with fasting triglycerides > 2.26 mmol/l.

## Statistical analysis

Sample size calculations were based on the need to ensure that the proportion of patients reporting on the primary and secondary endpoints could be estimated with sufficient precision, overall and on a by-sub-population basis. Hence, sample size determination was not based on test power considerations, but on the confidence limit approach to ensure adequate precision estimates.

For a precision within ± three percentage points, the length of the two-sided 95% confidence interval (CI) should not exceed three percentage points in each direction from the point estimate. For sample size calculations, and in accordance with standard practice, the proportion of patients achieving the LDL-C goal was assumed to be 50%, as this proportion was unknown. Based on these calculations, the minimum sample size of about 1 000 patients was considered sufficient to meet the primary endpoint for the smallest sub-population, patients with the metabolic syndrome.

Approximately 1 060 000 patients in South Africa are treated with statins, of which 850 000 (80%) are treated as primary prevention and 210 000 (20%) as secondary prevention. It was assumed that in South Africa the proportion of patients with the metabolic syndrome was similar to the proportion of secondary prevention patients at around 35%. Based on these assumptions a total sample size of about 3 000 patients was considered necessary.

Analyses of primary and secondary endpoints were performed using the same models.

For each patient, the risk category was determined and a dichotomous variable was calculated indicating whether the patient had achieved the target LDL-C goal corresponding to the relevant risk category. The number and percentage of patients achieving the LDL-C goals according to the NCEP ATP III/2004 updated NCEP ATP III/Fourth JETF/South African guidelines are presented.

Furthermore, a two-level logistic regression analysis was performed to determine the prognostic factors of achieving the LDL-C goals, according to each of the guidelines, with patients at the first level and physicians at the second level. Prognostic factors were identified among several patient and physician independent variables.

First the crude (univariate) association of each of the potential predictors with the outcome (i.e. achievement of LDL-C goals according to the corresponding guideline) was investigated as a univariate analysis using logistic regression. The dependent variable was achievement of the LDL-C goals according to the corresponding guidelines (yes or no), the potential predictor was fixed effects and the physician was random effect.

The association of fixed effects with the dependent variable was appraised by estimated odds ratio with associated 95% CIs and *p*-values. All predictors with a *p*-value < 0.10 (using the Wald-type test) in this crude (univariate) association analysis were further included in an adjusted multiple logistic regression model.

Discrete variables with more than two categories were fitted in the model using SAS PROC GENMOD through a series of binary variables by means of the CLASS statement. In cases where too small a number of answers per category was observed, categories were pooled.

First, a full model of random intercept variables selected based on the univariate analysis as fixed effects was evaluated. At each subsequent step, the least significant independent variable was removed until all variables reached a level of significance of at least 0.05.

For the final model, the following results were provided: parameter estimates and 95% CIs anti-logged to obtain the odds ratio and 95% CI of the odds ratio, standard error and *p*-value for each effect. The type III effects Wald-type test was used to assess the significance of a variable.

## Results

## Baseline characteristics

In total, 3 001 patients consented to participate in the survey. Laboratory data were missing for three patients and two patients withdrew their consent. Therefore the full analysis set (FAS) comprised 2 996 patients, recruited at 69 study centres. Demographic characteristics of this survey cohort are summarised in Table 2. The mean age was 59.4 years and 47.5% of the patients were female. A medical history of arterial hypertension, diabetes and CHD was reported by 71.6, 47.1 and 35.4% of the patients, respectively.

**Table 2.** Summary Of Demographics And Patient Characteristics

*Patient characteristics*	*Study cohort (n = 2 996)*
Age (years)*	59.4 (11.4)
Gender	
Male	1572 (52.5)
Female	1424 (47.5)
Ethnic group	
Caucasian	1385 (46.2)
Non-Caucasian	1611 (53.8)
Black	510 (17.0)
Mixed ancestry	481 (16.1)
Indian	576 (19.2)
Asian	44 ( 1.5)
BMI (kg/m^2^)*	30.0 ( 6.0)
Waist circumference (cm)*	101.0 (14.1)
SBP (mmHg)*	133.2 (17.7)
DBP (mmHg)*	80.2 ( 9.9)
Current smoker	445 (14.9)
Diagnosed diabetes	1411 (47.1)
Undiagnosed diabetes	71 ( 2.4)
Diabetes and history of coronary heart disease	494 (16.5)
Arterial hypertension	2144 (71.6)
Family history of premature CVD	863 (28.8)
History of coronary heart disease	1060 (35.4)
History of peripheral artery disease	146 ( 4.9)
History of cerebrovascular atherosclerotic disease	158 ( 5.3)

Results expressed as *n* (%) except where indicated by an asterisk (*) where reported as mean (standard deviation) CVD: cardiovascular disease, BMI: body mass index, SBP: systolic blood pressure, DBP: diastolic blood pressure, age (years): calculated relative to the subject’s date of visit, percentages are calculated relative to the total number of subjects with data.

When patients were asked about the measures taken by their physician when they were first diagnosed with high cholesterol levels, 64.2% had been prescribed lifestyle changes and pharmacotherapy, 20.2% had been prescribed pharmacotherapy only, 14.4% reported having been advised lifestyle modifications alone, and 1.2% had received neither pharmacotherapy nor lifestyle modification advice.

At the time of their assessment, most patients (95.9%) were receiving LLD monotherapy, with the majority (98.9%) receiving statins. Simvastatin, atorvastatin and rosuvastatin were the statins most frequently used in monotherapy (53.5, 30.3 and 12.9%, respectively). All of the patients taking multiple LLDs received statins in combination with other drugs, mainly fibrates (64.2%). Primary CVD prevention was the main reason for LLD therapy (44.3% of patients).

In total, 149 investigators were eligible to participate in the physician survey, 120 of them completed and returned the investigator’s questionnaire, while 101 investigators both returned the completed investigator’s questionnaire and examined patients. Most were male (80.2%) and general practitioners/primary care practitioners (GP/PCP) (66.3%); only 12.9% were cardiologists, 6.9% endocrinologists and 13.9% other specialist physicians.

**Fig. 1.**
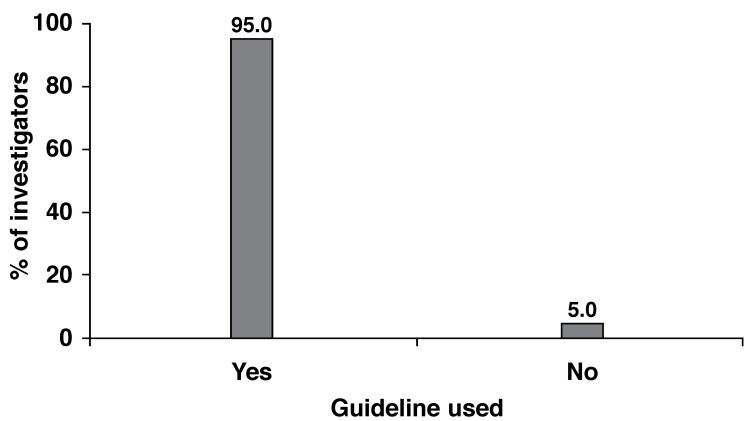
Use of guidelines to establish individual target cholesterol levels.

**Fig. 2.**
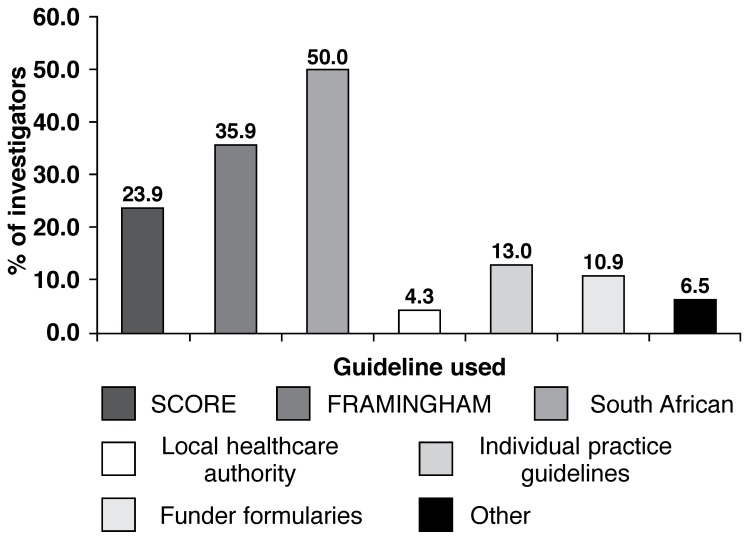
Guidelines used to establish cholesterol targets.

Most investigators (*n* = 96; 95.0%) use guidelines to establish individual target cholesterol levels, with the National South African guidelines and the NCEP ATP III guidelines (Framingham) being the most frequently used by 50.0% and 35.9% of investigators, respectively (Fig. 1). Other less-used guidelines were: Joint European guideline (SCORE) (*n* = 22; 23.9%), individual practice guidelines (*n* = 12; 13.0%), funder formularies (*n* = 10; 10.9%), other guidelines (n = 6, 6.5%) and local healthcare authority guidelines (*n* = 4; 4.3%) (Fig. 2).

## Lipid values and attainment of guideline targets

The overall mean plasma lipid and glucose values are presented in Table 3. The mean values of total cholesterol and LDL-C were 4.88 mmol/l and 2.73 mmol/l, respectively (Table 3).

**Table 3.** Summary Of Laboratory Results

*Test*	*Study cohort (n = 2996) Mean (SD)*
Total cholesterol (mmol/l)	4.88 (1.24)
Triglycerides (mmol/l)	1.90 (1.71)
HDL-C (mmol/l)	1.31 (0.39)
LDL-C (mmol/l)	2.73 (1.01)
Non-HDL-C (mmol/l)	3.58 (1.22)
Apo A1 (g/l)	1.39 (0.31)
Apo B (g/l)	0.87 (0.27)
Apo B/Apo A1 ratio	0.70 (0.30)
Glucose (mmol/l)	6.63 (2.95)
With diagnosed diabetes (n = 1 411)	8.16 (3.59)
HbA_1c_ (%)	7.11 (1.96)
With diagnosed diabetes (n = 1 411)	8.33 (2.19)

SD: standard deviation, HDL-C: high-density lipoprotein cholesterol, LDL-C: low-density lipoprotein cholesterol, ApoA1: apolipoprotein A1, ApoB: apolipoprotein B, HbA_1c_: haemoglobin A_1c_. Non-HDL-C (mmol/l): [total cholesterol (mmol/l) – HDL-C (mmol/l)].

## Target attainment according to the NCEP/2004 NCEP ATP III guidelines

Overall, 60.5% (*n* = 1 801) of patients with no missing data (*n* = 2 976) were at target LDL-C goals as recommended by NCEP ATP III/2004 NCEP ATP III guidelines (Table 4).

**Table 4.** Patients Achieving The LDL-C Goals Recommended By The Different Guidelines

	*NCEP ATP III/2004 NCEP ATP, n (%)*	*European/South African guidelines n (%)*
Overall survey	1801 (60.5)	1557 (52.3)
Age (years)		
< 40	61 (42.4)	52 (36.1)
40–54	441 (56.0)	382 (48.5)
55–69	941 (62.4)	822 (54.5)
≥ 70	358 (66.8)	301 (56.2)
Gender		
Male	992 (63.6)	854 (54.7)
Female	809 (57.1)	706 (49.6)
Body mass index (kg/m^2^)		
Normal weight (< 25 kg/m^2^)	352 (61.6)	293 (51.3)
Overweight (25–29 kg/m^2^)	667 (61.7)	584 (54.0)
Obese (≥ 30 kg/m2)	780 (59.1)	678 (51.4)
Coronary heart disease	591 (56.0)	532 (50.4)
Peripheral artery disease	77 (52.7)	69 (47.3)
Cerebrovascular atherosclerotic disease	91 (58.7)	81 (52.3)
Current smoker	234 (52.9)	213 (48.2)
Diabetes	837 (59.6)	764 (54.4)
Arterial hypertension	1290 (60.6)	1136 (53.4)
Family history of premature cardiovascular disease	462 (53.5)	391 (45.3)
Type of prevention		
Primary prevention	882 (67.0)	722 (54.8)
Secondary prevention	492 (56.9)	437 (50.6)
Diabetes mellitus	388 (58.2)	364 (54.6)
Familial hypercholesterolaemia	39 (30.2)	34 (26.4)
Metabolic syndrome (Alberti *et al.* 2009)	1177 (58.2)	1031 (50.9)
Type of therapy		
Statin monotherapy	1720 (61.3)	1485 (53.0)
Fibrates monotherapy	9 (36.0)	7 (28.0)
Combination therapy	59 (48.8)	53 (43.8)
Risk category		
High risk	1201 (55.5)	1086 (50.2)
Medium/low risk	600 (73.8)	471 (57.9)

The level of achievement of LDL-C targets improves as the patient ages (< 40 years: 42.4%; 40 to < 55 years: 56.0%; 55 to < 70 years: 62.4%; ≥ 70 years: 66.8%). More males (63.6%) reached the LDL-C goals than females (57.1%). Achievement of LDL-C goals was not affected by BMI, with the respective at-goal rates of 61.6% in normal-weight patients, 61.7% in overweight and 59.1% in obese patients.

Stratification according to LLD showed that 61.3% of patients on statin monotherapy, 48.8% on combination therapy and 36.0% on monotherapy with fibrates achieved their LDL-C goals. In patients on statin therapy, more achieved their goals on the more potent statins (Rosuvastatin and Atorvastatin) compared to those on the less potent statins (Table 5).

**Table 5.** Patients On Statins Achieving The LDL-C Goals

	*Atorvastatin (n = 866)*	*Fluvastatin (n = 10)*	*Lovastatin (n = 4)*	*Pravastatin (n = 43)*	*Rosuvastatin (n = 371)*	*Simvastatin (n = 1 526)*
Mean dosage (mg)	20.6	44.0	17.5	23.6	14.7	21.7
Controlled LDL-C NCEP*	62%	60%	25%	44%	71%	59%
Controlled LDL-C Eu/SA*	53%	30%	25%	33%	61%	52%

* Chi-square test; *p* < 0.05

Differences were noted in the achievement of LDL-C goals in different ethnic groups, with Asians showing the highest achievement (65.9%), followed by Caucasians (64.9%), blacks (63.3%), Indians (55.5%), and the mixed-ancestry (50.6%) group.

The percentage of patients who attained the LDL-C goal was highest among primary-prevention patients (67.0%), similar among patients treated for diabetes mellitus (58.2%) and in secondary prevention after a cardiovascular event (56.9%), but lowest among patients with familial hypercholesterolaemia (30.2%). Only 58.2% of patients with the metabolic syndrome achieved the LDL-C goal, while 65.5% of patients without the metabolic syndrome achieved the LDL-C goal.

## Target attainment according to the Fourth JETF/South African guidelines

Only 52.3% (*n* = 1 557) of patients with no missing data (*n* = 2 976) reached the LDL-C goals recommended by the Fourth JETF/South African guidelines (Table 4). The percentage of patients reaching the LDL-C goals was higher in male patients (54.7%) than in females (49.6%). Younger patients achieved LDL-C goals less frequently, with only 36.1% of patients under 40 years and 48.5% of patients aged 40 to 54 years reaching the LDL-C goal, while 54.5 and 56.2% reached goal in the 55 to 69 years and ≥ 70 years categories, respectively.

A similar percentage of patients reaching the LDL-C goals was observed in normal-weight (51.3%), overweight (54.0%) and obese (51.4%) patients. The percentage of LDL-C goal achievers was slightly lower in patients with CHD (50.4%) than in those without CHD (53.4%), and similar in patients with (52.3%) and without (52.3%) CAD.

Conversely, more patients without peripheral arterial disease (PAD) (52.6%) reached the LDL-C goals versus patients with PAD (47.3%). Fewer patients with a family history of premature CVD (45.3%) reached the LDL-C goal versus those without a family history of premature CVD (55.2%). More patients with diagnosed diabetes (*n* = 1 411; 54.4%) achieved the LDL-C goal than patients with probable undiagnosed diabetes based on a single fasting glucose of > 7 mmol/l (*n* = 71; 40.8%).

More patients on statin monotherapy (53.0%) or combination therapy (43.8%) reached their LDL-C goal, compared with patients on monotherapy with fibrates (28.0%). Differences were noted in the achievement of LDL-C goals in different ethnic groups, with Asians showing the highest achievement (59.1%), followed by blacks (58.0%), Caucasians (54.5%), Indians (49.4%), and the mixed-ancestry (43.1%) group.

More patients who were non-smokers (53.0%) achieved LDL-C goals than patients who were smokers (48.2%). The percentage of patients who were at the LDL-C recommended level was 54.9% for those in primary prevention, 54.6% for those in secondary prevention and 26.4% for those with familial hypercholesterolaemia.

The percentage of patients with the metabolic syndrome who achieved their LDL-C goal was 50.9%. When stratified according to LLD, 53.0% of patients on monotherapy with statins, 53.0% of patients on combined therapy, and 28.0% of patients on monotherapy with fibrates achieved their LDL-C goals. The percentage of patients at the LDL-C goal recommended by the Fourth JETF/South African guidelines in different sub-populations is also summarised in Table 4.

## Determinants for attainment of the NCEP and Fourth JETF/South African guidelines targets

**Fig. 3.**
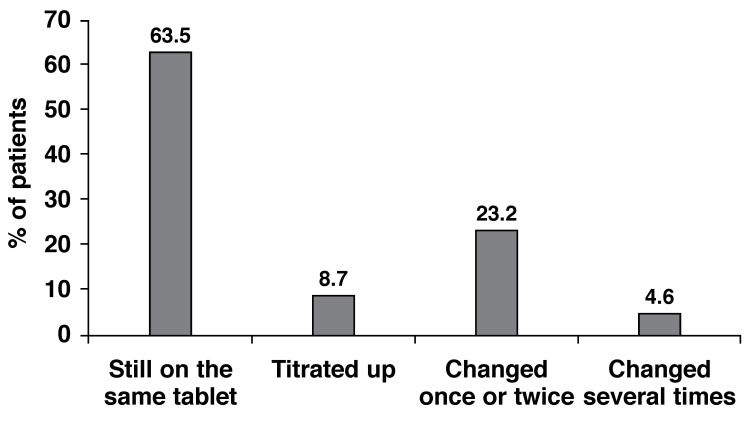
Changes in LLD since first prescribed a drug.

A total of 72.2% of patients were still on the same LLD as when first prescribed lipid-lowering pharmacotherapy; 63.5% of all patients were still taking the initial starting dose, while the dose had been increased in 8.7% of patients. In 23.2% of patients the LLD had changed once or twice, while in 4.6% of patients the LLD had changed several times (Fig. 3).

A total of 63.5% of patients were still on the same LLD as when first prescribed lipid-lowering pharmacotherapy; 63.5% of all patients were still taking the initial starting dose, while the dose had been increased in 8.7% of patients. In 4.6% of patients, the LLD had been changed several times.

**Fig. 4.**
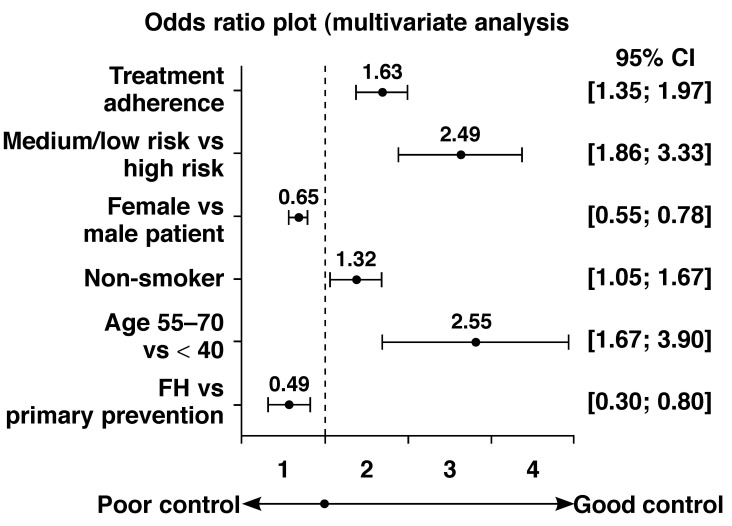
Significant multivariate predictors of reaching the NCEP LDL-C target levels.

Patient predictors that significantly influenced achieving of LDL-C goals recommended by the NCEP ATP III/2004 updated NCEP ATP III guidelines included being older in age; male gender; being a non-smoker; not having familial hypercholesterolaemia; being in the medium/low-risk category rather than high risk; and compliance with treatment, including compliance after cholesterol had returned to normal levels (Fig. 4).

Predictors that significantly influenced being at the LDL-C goal according to the Fourth JETF/South African guidelines included being older in age, male gender, having diagnosed diabetes, not having a family history of premature CVD, being medium/low risk rather than in the high-risk category, and compliance with treatment, including compliance after cholesterol returned to normal.

## Discussion

Cardiovascular disease is the leading cause of mortality and morbidity in adult patients in industrialised countries. The relationship between increased LDL-C levels and increased risk of CHD is widely accepted, as is the corollary that lowering LDL-C levels reduces CHD mortality. Hence, most practice guidelines for CVD management focus on LDL-C as the primary target.

All guidelines encourage physicians to initially focus on patients with established atherosclerotic disease as these stand to gain the largest absolute short-term benefit, but preventive measures in healthy individuals with a high global risk of CHD (i.e. primary prevention) should also not be neglected.^16^,^17^

The results of this survey indicate that most physicians are aware of the current guidelines and that 95.0% of physicians use them to set individual cholesterol targets for their patients. Among all guidelines, the most frequently used was the National South African guidelines (50.0%), followed by the NCEP ATP III guidelines (35.9%) and the JETF guidelines (23.9%).

Despite the widespread awareness of recent guidelines, the results of this survey indicate that the control of hypercholesterolaemia is sub-optimal. As highlighted by the EUROASPIRE surveys^11^,^12^ conducted in multiple European countries, the CEPHEUS SA survey confirms that control of modifiable risk factors remains sub-optimal in many patients at high cardiovascular risk. For instance, obesity at 44.5% and smoking at 14.9% remained highly prevalent. These results do not significantly differ from those obtained in the two EUROASPIRE surveys,^18^,^19^ and for certain modifiable factors, their current prevalence is even higher.

The results of the current survey indicate that a large proportion of patients on lipid-lowering pharmacological therapy in South Africa failed to achieve their cholesterol goal levels. Only 60.5 and 52.3% of patients on LLD for at least the previous three months attained the LDL-C target level recommended by the 2004 updated NCEP ATP III guidelines and the lower target proposed by the JETF/South African guidelines.

In agreement with the published literature, the percentage of patients attaining the LDL-C goal was higher among patients treated with statins. Statins have been shown to improve cardiovascular outcomes in a wide range of patients and are the most powerful LDL-C-lowering drugs currently marketed.^20^,^21^

The poor goal-achievement rates observed in the current survey do not seem to be correlated with a poor awareness of hypercholesterolaemia among patients. Most patients (73.3%) were informed of their cholesterol level and had been given a cholesterol target (80.4%). Most patients were aware of the role of both LDL-C and HDL-C in CVD risk, and were satisfied with the level of information provided by their physicians. Nonetheless, the level of knowledge of the general public regarding dyslipidaemia seems to be much lower. In the REACT survey,^22^ physicians claimed that 92% of their patients knew about LDL-C and HDL-C, but when members from the general public were interviewed, only 23 and 25% of them were aware of the importance of LDL-C and HDL-C, respectively. It is assumed that in South Africa a large percentage of the population do not know their cholesterol levels.

Although 60% of the patients have been reported to stop taking their lipid-lowering medication once they reach their cholesterol target in a prospective survey in Australia,^23^ in this survey which enrolled patients attending mainly for review of lipid-lowering therapy, only 4% of the interviewed patients acknowledged stopping their lipid-lowering therapy once they had reached their goal.

## Limitations

The present study has some limitations. Firstly, the population was a selected group of patients treated with LLD, and the findings cannot be extrapolated to the general population. More motivated patients may have been more likely to agree to participation in the survey, introducing a potential positive bias. The same bias could apply to the participating physicians. It is possible that not all patients completed the questionnaire truthfully, but rather chose answers they thought would please their treating doctor. CEPHEUS SA was performed as a survey, without independent verification of the answers provided. The questionnaires were not validated, but only used for exploratory purposes.

The diagnosis of metabolic syndrome was made using treated lipid values. This may have resulted in some patients with the metabolic syndrome being missed, as LLDs can lower triglyceride and increase HDL-C levels. Untreated values for these lipid variables were generally not available. Finally, caution is needed when interpreting correlations, since causal relationships were not established.

## Conclusions

Overall, the results of this survey highlight the sub-optimal lipid control achieved in many South African patients prescribed LLDs. Poor goal attainment may result from many factors. Patient factors may include inadequate lifestyle modification, sub-optimal adherence to medication and poor long-term persistence with therapy. Many of these factors are modified or determined by the patient’s attitude to taking medication (e.g. concerns about safety and toxicity) and the perceived benefit of lipid-lowering medication, which tends to be highest in those with previous cardiovascular events. High out-of-pocket costs may also affect adherence negatively.

Physician factors may include unawareness of lipid targets, unwillingness to treat to target due to concerns about drug toxicity, drug formulary constraints, and failure to check lipid levels once LLDs have been started, and to titrate LLD dosage if required. Patients with severe genetic dyslipidaemias such as familial hypercholesterolaemia frequently do not reach lipid goals, as currently available LLDs are not sufficiently potent. Some patients may be unable to tolerate high doses of LLDs and therefore fail to reach goal.

Poor lipid goal attainment therefore is a complex problem that cannot be resolved by a single across-the-board intervention. Physician education and incentivisation may address some of the physician-related factors leading to poor lipid control, while patient-related factors can only be addressed at an individual level. Availability and affordability of lipid-lowering medication remains problematic for both private and public sector patients in South Africa.
